# A young man with recurrent kidney stones and renal failure 

**DOI:** 10.5414/CNCS110198

**Published:** 2020-11-03

**Authors:** Jasmeet Gill, Michael R. Wiederkehr

**Affiliations:** 1 Baylor University Medical Center, Division of Nephrology, Department of Internal Medicine, and; 2 Texas A&M Health Science Center College of Medicine, Dallas Campus, Dallas, TX, USA

**Keywords:** kidney stones, nephrocalcinosis, genetic, low molecular weight proteinuria, Dent, CLCN5

## Abstract

Dent disease is an inherited proximal renal tubulopathy leading to low molecular weight proteinuria, hypercalciuria with nephrocalcinosis and nephrolithiasis, and progressive renal failure. Two genetic mutations have been identified. The disease usually presents in childhood or early adult life and may be associated with other proximal tubular defects, which can lead to significant morbidity, especially in children. The disorder can extend to interstitial and glomerular cells, which contributes to progression to end-stage kidney disease. The pathophysiologic process remains incompletely understood, and no specific treatment is available. Dent disease is likely under-recognized. It needs to be included in the differential, especially in young males, presenting with recurrent kidney stones, proteinuria, and impaired renal function.

## Introduction 

Nephrolithiasis is a common disease with a prevalence of ~ 10% in the general population in the United States, of which ~ 50% will have recurrence at 5 – 10 years [1]. Early onset of stones, a high stone burden, a positive family history, and association with extra-renal manifestation (hearing defects, ocular abnormalities, and others) suggest a possible genetic cause. The five main causes of inherited metabolic disorders leading to renal stones are the following: adenine phosphoribosyl transferase (APRT) deficiency, cystinuria, Dent disease, familial hypomagnesemia with hypercalciuria and nephrocalcinosis (FHHNC), and primary hyperoxaluria (PH). Patients typically experience recurring stones starting in childhood and are at risk of developing chronic kidney disease (CKD) and even progression to end-stage kidney disease. 

Dent disease is a rare, X-linked recessive disorder that presents with the classic triad of low molecular weight (LMW) proteinuria, hypercalciuria with nephrocalcinosis, and nephrolithiasis mainly consisting of calcium oxalate and calcium phosphate stones. LMW proteinuria is a clinical hallmark that is present in almost all affected males and obligate female carriers. Dent and Friedman first described the disease in 1964 in two unrelated English boys who presented with rickets, acidosis, and hypercalciuria [2]. Prior to the discovery of the genetic mutation, various terminologies were used to describe Dent disease, including, in North America, X-linked recessive nephrolithiasis with renal failure, X-linked recessive hypophosphatemic rickets in Europe, and idiopathic LMW proteinuria in Japan [3]. 

Dent disease is a heterogeneous group of disorders, but the majority of patients have either one of two genetic mutations, and it may overlap with another genetic disorder, Lowe syndrome [4, 5]. Its initial presentation is nonspecific, and thus rarely considered in the evaluation of a patient with proteinuria, kidney stones and/or nephrocalcinosis, and an unclear cause of renal failure. 

## Case presentation 

A 46-year-old Caucasian man presented with recurrent kidney stones and worsening renal failure. The presence of proteinuria was first documented at the age of 18 years during a routine physical screening for military service; however, this did not lead to further evaluation. At age 30, he was evaluated for non-nephrotic proteinuria and an elevated serum creatinine in the range of 1.7 – 2.0 mg/dL. His serum calcium, phosphorus, and parathyroid hormone levels were normal. He did not have hypertension, diabetes mellitus, or gout. In fact, he had borderline hypotension and did not tolerate treatment with ACE inhibitor or angiotensin-2 receptor blocker. He did have recurrent nephrolithiasis, with stone passage since his mid-twenties. A stone analysis at that time showed 65% calcium oxalate and 35% calcium phosphate. His surgical history was negative except for lithotripsy once. His medications included allopurinol, hydrochlorothiazide, and potassium citrate. He was married and had one healthy 12-year-old daughter. He never smoked. His parents were alive and well without kidney disease or kidney stones. To the best of his knowledge, his grandparents had no kidney-related diagnoses. However, his sister’s children had developed kidney stones during their teenage years. His renal ultrasound revealed small kidneys at 9 – 10 cm with thin cortices and nephrocalcinosis, and small cysts. Additionally, multiple small bilateral calculi were demonstrated on CT scan. Kidney biopsy showed 10 glomeruli, of which 2 were globally sclerosed. The viable glomeruli showed an increase in mesangial matrix and endocapillary cells. The capillary walls were normal, thin, and widely patent. There was interstitial edema and focal interstitial fibrosis. The arteries, arterioles, and tubules were unremarkable. Electron microscopy revealed normal capillary basement membranes without deposits, a mild increase in mesangial matrix with some areas of small electron-dense deposits. The epithelial cells were markedly swollen with patchy foot process fusion. Immunofluorescence stains with adequate controls showed no IgG, IgM, or C3 deposits, and only trace and patchy granular mesangial staining for IgA. Overall, the changes were felt to be non-specific and could not be categorized to a specific diagnosis. 

On presentation to our clinic, he was a well-developed muscular man with normal stature, and normal vital signs. His physical exam was unrevealing; laboratory values are outlined in Tables 1, 2, and 3. He was at CKD stage 4 with a creatinine of 2.8 mg/dL corresponding to an estimated glomerular filtration rate (eGFR) of 26 mL/min/1.73m^2^ (CKD-EPI). Electrolytes were normal, including normal serum phosphorus and parathyroid hormone levels. A urine dipstick indicated urine pH of 7.0 without glycosuria but with occult blood and albumin, and microscopic exam revealed 5 – 10 non-dysmorphic red blood cells, a few fine granular casts, and copious amorphous sediment (Tables 2, 3). 

A stone analysis in our clinic showed 90% calcium oxalate monohydrate, and 10% calcium phosphate. We discontinued allopurinol but continued hydrochlorothiazide 12.5 mg daily, and potassium citrate 10 mEq twice daily. On this regimen, a 24-hour urine stone profiling showed an alkaline urine pH at 7.1 and borderline hyperoxaluria and mild hypocitraturia (Table 4). 

Urine protein analysis determined a protein-to-creatinine ratio of 1,010 mg/g with an albumin-to-creatinine ratio of 208 mg/g, indicating a significant proportion of non-albumin proteinuria. Evaluation for LMW proteinuria indicated a urine β-2 microglobulin level at a very high level at 78,705 µg/L (reference range 0 – 300 µg/L), or 107 mg/g creatinine. Subsequent genetic analysis was positive for *CLCN5* mutation consistent with Dent 1 disease. 

We provided dietary advice and continued our current treatment regimen, and his renal function remained stable with some fluctuations around an eGFR of 25 mL/min/1.73m^2^ during the 2.5 years of follow-up in our clinic. The patient subsequently moved out of state, but we were able to trace his renal function over a time period of 22 years (1998 – 2020), which showed the expected slow but insidious decline in renal function. On last follow-up on July 20, 2020, his creatinine had increased to 3.2 mg/dL (eGFR 21 mL/min/1.73m^2^), and first discussions about renal transplantation and the possibility of renal replacement therapy were initiated. 

## Discussion 

Our patient presented with progressive CKD with non-nephrotic range proteinuria, nephrocalcinosis with recurrent nephrolithiasis, a non-specific kidney biopsy, and positive family history for kidney stones starting in late childhood. This was suggestive for a genetic cause of nephrolithiasis. On subsequent evaluation for LMW proteinuria, specifically β-2 microglobulin, this was shown to be ~ 500-fold above the upper limit of normal, and our suspicion for Dent disease was confirmed by genetic testing. 

Dent disease (Online Mendelian inheritance of Man (OMIM) 300009) is a rare X-linked recessive proximal tubular disorder, although the recessive inheritance pattern has been disputed. The large variations of penetrance of the genotype in females are due to multiple mechanisms on a cellular level (cell autonomous expression, skewed X inactivation, clonal expansion, somatic mosaicism) that do not reconcile with the standard definition of a recessive inheritance pattern and can lead to a highly variable phenotype of an X-linked disorder in females, as described in detail elsewhere [6]. However, in general, the classic phenotype of the disease is present in males only, while females are carriers. 60% of patients with Dent disease have a mutation in the *CLCN5* gene (Dent-1), while only 15% have a mutation in the *OCRL1* gene (Dent-2), and both mutations are located on the X chromosome. In the remaining 25%, the specific gene defects are not known [7]. 

Our patient reported that his sisters’ two sons developed kidney stones during their teenage years, and indeed, one of them had Dent disease confirmed by genetic testing. As mentioned, neither of the patient’s parents had kidneys stones, nor did his maternal grandfather. Therefore, it is safe to assume that both his mother and his maternal grandmother were asymptomatic carriers for the disease. 

The pathophysiology of Dent remains incompletely understood. The chloride channels (CLC) are a group of ion-specific channels and transporters associated with a variety of diseases, including neuromuscular (CLC1: myotonia congenital), bone (CLC7: osteopetrosis), and kidneys (CLCKB: Bartter disease; CLC5: Dent-1). Malfunction of the chloride channel CLC5 probably affects the electrical gradient in the proximal tubular cell, which impairs the function of a receptor-mediated endocytic pathway involving the megalin-cubulin system, required for the absorption of LMW proteins. Likewise, OCRL1 is also involved in the renal tubular endocytic pathway [8]. 

The *CCLN5* gene encodes for a voltage-gated chloride channel in proximal tubular cells. Approximately 150 different types of CCLN5 mutations are documented, including nonsense, missense, and frame shift mutations, producing truncation of the protein and loss of function. The *OCRL1* gene encodes phosphatidylinositol 4,5-biphosphate 5-phosphatase localized at the Golgi apparatus. *OCRL1* mutations are also described with Lowe syndrome, which also features mental retardation, growth retardation, and congenital cataract [7] – not present in Dent. To date, 250 affected families of Dent-1 and 25 Dent-2 families have been reported [9]. 

The pathophysiologic process leading to hypercalciuria is at best speculative. The megalin-cubilin complex is involved in the reabsorption of filtered parathyroid hormone (PTH). The defect in Dent disease leads to high urinary levels of PTH, which may increase activity of the 1-α hydroxylase enzyme in the proximal tubule and subsequently increase intestinal absorption of calcium with hypercalciuria via enhanced levels of 1, 25 (OH) vitamin D3 [9]. The stones in Dent disease are typically composed of both calcium oxalate and calcium phosphate as in our patient, because of both hypercalciuria, and a urinary acidification defect (our patient’s urinary pH was 7.1). Nephrocalcinosis is commonly observed and may contribute to the progression of renal disease, although other factors are likely involved, including glomerular disease. Review of the Rare kidney Stone Consortium (RKSC) registry data revealed that on kidney biopsy, 83% of patients had focal global glomerulosclerosis, and ~ 60% of the biopsies had mild segmental foot process effacement and focal interstitial fibrosis [10]. Our patient’s kidney biopsy in fact did show glomerular sclerosis and focal interstitial fibrosis with patchy foot process effacement. Dent disease should be considered in boys and men with unexplained proteinuria with focal global glomerulosclerosis and segmental foot process effacement on renal biopsy. 

Clinically, the universal findings are elevated levels of urinary LMW proteins, typically α-1 microglobulin, β-2 microglobulin, and retinol-binding protein, usually 100 to 1,000 times above the upper range of normal. Other markers of proximal tubular dysfunction, such as aminoaciduria, phosphaturia with hypophosphatemia, glycosuria, and an alkaline urinary pH, are less consistent [5, 11]. Our patient had the very high level of β-2 microglobulin and the alkaline urine pH of 7.1, but none of the other markers of proximal tubular dysfunction. 

Most male patients present with nephrocalcinosis with or without kidney stone passage, and progressive CKD. LMW proteinuria is the earliest and most consistent finding, β-2 microglobulin is the most frequently measured LMW protein and useful to screen for the disease. Our patient had proteinuria of 1.2 g on initial presentation as well as an elevated creatinine, nephrocalcinosis, and nephrolithiasis. However, the proteinuria was not further evaluated as to the type of urinary protein (albumin or LMW protein). We believe that the identification of LMW proteinuria could have been an early diagnostic clue for Dent disease. Of course, in our particular patient, the positive family history for early kidney stones in his nephews also suggested a genetic cause. 

**Figure 1.**
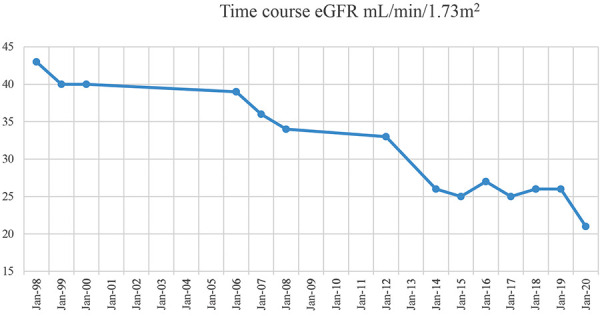
eGFR time course of our patient.

The progression of CKD is variable, but most males reach end-stage kidney disease between the ages of 30 and 50 years. The rate of decline in renal function correlates with the degree of proteinuria, glomerular damage, and severity of interstitial fibrosis [12]. Our patient is now 52 years old and has shown the typical decline in eGFR, to a most recent serum creatinine of 3.2 mg/dL (eGFR 21 mL/min/1.73m^2^) on July 20, 2020, and thus has not yet reached “end-stage” (Figure 1). 

A patient presenting with hypercalciuria, nephrocalcinosis, and recurrent nephrolithiasis with progressive CKD has broad differentials, that include defects causing proximal tubule dysfunction like cystinosis, galactosemia, idiopathic hypercalciuria, and medullary sponge kidney, also rarer causes such as distal renal tubular acidosis, familial hypomagnesemia with hypercalciuria, and nephrocalcinosis as well as Bartter’s syndrome [8]. In the pediatric population presenting with rickets and osteomalacia, both inherited and acquired causes of Fanconi syndrome need to beconsidered. Dent disease classically presents with LMW proteinuria, progressive renal disease, and rarely with classical Fanconi syndrome. However, there have been ~ 100 patients reported with incomplete Fanconi’s presenting with hyperuricosuria, hyperphosphaturia, and aminoaciduria [14]. Our patient only had slightly elevated hyperuricosuria at 816 mg per day, but a normal serum uric acid level and no hyperphosphaturia or glycosuria. As discussed above, kidney biopsy findings are non-specific, showing focal global or segmental glomerulosclerosis, tubular atrophy, interstitial inflammation, and interstitial fibrosis [13]. The clinical picture with suggestive X-linked inheritance and genetic identification of *CCLN5* or *OCRL1* mutation confirm the diagnosis. 

Currently there is no specific treatment targeting the molecular defect in Dent disease. The goal of treatment is to delay the progression of CKD and to prevent nephrolithiasis. Thiazide type diuretics aide in reducing hypercalciuria and the formation of kidney stones. Inhibitors of the renin-angiotensin-aldosterone system are often used although there is no outcome data. Electrolyte abnormalities like hypokalemia and hypophosphatemia require supplementation. Patients with Dent disease are good candidates for renal transplantation. 

Dent disease is likely under-diagnosed due to lack of awareness. The diagnosis can be challenging, as patients may present with clinically less overt forms, or with a negative family history. Interestingly, even within the same family, the phenotypic expression or penetration of the genetic mutation often varies significantly, with some family members lacking proteinuria, nephrocalcinosis, or abnormal renal function, despite the same genetic mutation [15]. A young patient may simply present with hypercalciuria and a history of calcium oxalate or calcium phosphate stones, and genetic testing is the key to confirm the diagnosis. 

In conclusion, our case report indicates that in male patients with CKD of unknown etiology along with LMW proteinuria, hypercalciuria, and nephrocalcinosis, a diagnosis of Dent disease should be in the differential. 

## Funding 

The authors received no financial support for publication of this article. 

## Conflict of interest 

The authors declare no financial conflict of interest in any of the products mentioned. 

**Table 1.** Laboratory findings of our patient.

Serum chemistries		Reference range
BUN	**25 mg/dL**	6 – 24
Creatinine	**2.79 mg/dL**	0.76 – 1.27
Sodium	139 mEq/L	134 – 144
Potassium	3.5 mEq/L	3.5 – 5.2
Chloride	96 mEq/L	97 – 108
Bicarbonate	28 mEq/L	18 – 29
Total protein	6.9 g/dL	6.0 – 8.5
Albumin	4.5 g/dL	3.5 – 5.5
Calcium	9.6 mg/dL	8.7 – 10.2
Phosphorus	2.9 mg/dL	2.5 – 4.5
Alkaline phosphatase	86 mg/dL	39 – 117
Uric acid	8.1 mg/dL	3.7 – 8.6
Parathyroid hormone	45 pg/mL	15 – 65
Vitamin D, 25-hydroxy	**23.4 ng/mL**	30 – 100
Vitamin D, 1-25 di-hydroxy	24.9 pg/mL	19.9 – 79.3

Abnormal values in bold. BUN = blood urine nitrogen.

**Table 2.** Urinalysis.

Urinalysis	
pH	**7.0**
Specific gravity	1.020
Glucose	Negative
Ketones	Negative
Protein	100
Nitrite	Negative
Occult blood	**Moderate**
WBC esterase	**Small**
Bacteria	None

Significant findings in **bold**. WBC = white blood cell.

**Table 3.** Urine microscopic exam.

Urine sediment exam	
WBC/HPF	**2** – **5**
RBC/HPF	**5** – **10**
Bacteria	None
Casts/LPF	**Hyaline and fine granular**
Epithelial cells	Rare
Casts	**Copious amorphous sediment**

Significant findings in **bold**. WBC = white blood cell; HPF = high power field; RBC = red blood cell.

**Table 4.** 24-hours urinalysis.

24-h urine collection		Reference range
Volume	3.5 L	
pH	**7.1**	5.8 – 6.2
Calcium	223 mg/day	Males < 250
Citrate	**410 mg/day**	Males > 450
Uric acid	**816 mg/day**	Males < 800
Oxalate	**41 mg/day**	20 – 40
Magnesium	118 mg/day	30 – 120
Sodium	211 mmol/day	50 – 150
Potassium	89 mmol/day	20 – 100
Phosphorus	942 mg/day	0.6 – 1.2
Chloride	206 mmol/day	70 – 250
Ammonium	22 mmol/day	15 – 60
Sulfate	38 meq/day	20 – 80
UUN	8.70	6 – 14

Significant findings in **bold**. UUN = urine urea nitrogen.
